# Carrier transfer efficiency and its influence on emission properties of telecom wavelength InP-based quantum dot – quantum well structures

**DOI:** 10.1038/s41598-018-30950-4

**Published:** 2018-08-17

**Authors:** Wojciech Rudno-Rudziński, Marcin Syperek, Janusz Andrzejewski, Ernest Rogowicz, Gadi Eisenstein, Sven Bauer, Vitalii I. Sichkovskyi, Johann P. Reithmaier, Grzegorz Sęk

**Affiliations:** 1 Department of Experimental Physics, Faculty of Fundamental Problems of Technology, Wrocław University of Science and Technology, St. Wyspiańskiego 27, 50-370 Wrocław, Poland; 2 Department of Electrical Engineering and Russell Berrie Nanotechnology Institute, Technion-Israel Institute of Technology, Haifa, 32000 Israel; 3 Technische Physik, Institute of Nanostructure Technology and Analytics, CINSaT, University of Kassel, Heinrich Plett-Str. 40, D-34132 Kassel, Germany

## Abstract

We investigate a hybrid system containing an In_0.53_Ga_0.47_As quantum well (QW), separated by a thin 2 nm In_0.53_Ga_0.23_Al_0.24_As barrier from 1.55 µm emitting InAs quantum dots (QDs), grown by molecular beam epitaxy on an InP substrate. Photoreflectance and photoluminescence (PL) spectroscopies are used to identify optical transitions in the system, with support of 8-band kp modelling. The main part of the work constitute the measurements and analysis of thermal quenching of PL for a set of samples with different QW widths (3–6 nm). Basing on Arrhenius plots, carrier escape channels from the dots are identified, pointing at the importance of carrier escape into the QW. A simple two level rate equations model is proposed and solved, exhibiting qualitative agreement with experimental observations. We show that for a narrow QW the escape process is less efficient than carrier supply via the QW due to the narrow barrier, resulting in improved emission intensity at room temperature. It proves that with carefully designed energy level structure, a hybrid QW/QD system can be used as an active region in telecom lasers with improved efficiencies.

## Introduction

In spite of theoretically predicted and experimentally demonstrated advantages of quantum dot (QD) based lasers^1^, such as ultralow threshold currents^2,3^, better temperature stability^4–6^ and broad gain spectrum leading to wide spectral tuneability^7^, QD lasers still have not reached their full application potential, especially in the spectral range of telecommunication windows, at 1.3 or 1.55 µm, since they suffer from limited speed of modulation crucial for fast data transfer rates, caused by considerable population of hot carriers occupying QD excited states and even wetting layer (WL).

One of possible routes forward is the application of a tunnel injection (TI) scheme, where an additional quantum well (QW) is grown in vicinity of the QD layer, separated by a thin barrier enabling carrier tunnelling^8,9^. The proper design of TI structure requires that the ground state of the entire tunnel system (both electron and hole lowest energy levels) is constituted by states confined in the QD part in order to take advantage of the 3D carrier confinement. The QW and barriers, on the other hand, must be chosen in such a way as to efficiently provide cold carriers, especially electrons, to the QDs, meaning that the barrier should be thin enough and the material composition and thickness of the QW must result in the lowest lying level having energy not much above the QD electron ground state, preferably by multiple of longitudinal optical phonon energy. Such a QD/QW hybrid system has also been demonstrated as beneficial in other applications, such as e.g. ultrafast injection of polarized spins^10,11^, QD-based memories^12^, infrared photodetectors^13^ and quantum-dot-based quantum cascade lasers^14^.

Tunnel injection based lasers have been already realized in several material systems. As foreseen, they exhibit high speed modulation^15^, high power^16^, ultralow threshold current^17^ and broad tuneability in a comb scheme^18^. However, most of the work so far has been devoted to the In(Ga)As/GaAs model system, which has limited application potential due to emission around 1.1 μm, far from telecom relevant range, and to the demonstration of laser characteristics, without detailed analysis of underlying physical processes, which is necessary to bring hybrid QW/QD structures to the level of practical applicability. There are several articles concerning dynamic properties of TI system, but focused only on GaAs based strutures^19–21^.

Here, there are demonstrated comprehensive studies of temperature dependence of emission from a TI system based on InAs on InP quantum dots, emitting at 1.55 μm at room temperature, with an InGaAs QW and a thin 2 nm wide InGaAlAs barrier. The experimental data are supported by numerical band structure calculations of the confined states within multi-band k·p theory. Previous numerical analysis of temperature dependence of TI-based laser operating characteristics, provided by ref.^22^ for GaInAsP/InP material system, points at an important role of recombination in the QW which controls laser’s threshold current and T_0_. PL quenching has been also investigated in InGaAs/GaAs systems emitting at 1.1 μm^23,24^ and 1.3 μm^25^. However, there is only one report on 1.55 μm emitting InP-based structures^26^, covering quantum dashes (highly elongated dots), in contrast to much more symmetric dots investigated here. These dots are characterised by larger separation between confined levels, owing to smaller volume of a single dot and thus more suitable for precise energy level structure design required for efficient tunnel injection. Moreover, in ref.^26^ only the influence of barrier thickness is investigated. The present work focuses on the analysis of temperature dependence of emission efficiency, governed by the competing processes of carrier supply from the QW to the dots, which seems to be very effective due to narrow, only 2 nm wide barriers; and out-tunnelling of carriers from QDs to QW. This carrier escape channel efficiency strongly correlates with relative energy positions of confined levels in QW and QD subsystems (also confirmed by employing rate equations model), which is controlled by changing QW width and by temperature, due to the participation of phonons mediating the transfer process. With support of detailed and realistic calculations of the energy structure of the whole coupled QW/QD system, the analysis provided here sheds light on the practical applicability of TI scheme in lasers for the most relevant telecom wavelength range, showing that for an appropriately designed hybrid system a considerable improvement in room temperature PL intensity can be achieved for TI structure emitting at 1.55 μm.

## Results and Discussion

### Electronic structure

**Figure 1:**
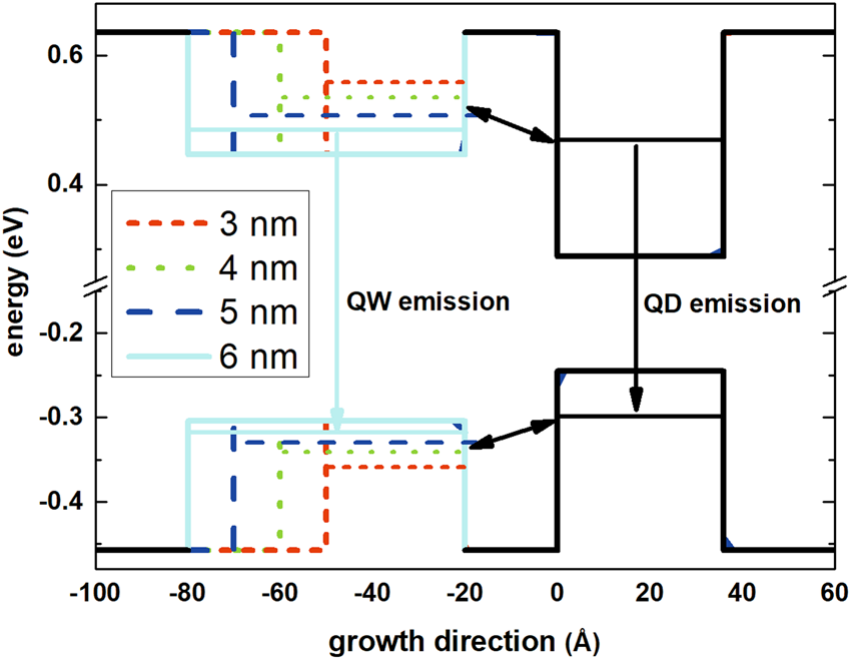
Conduction and valence band profiles for four investigated samples. Different line styles and colours indicate changing QW width. Horizontal line denote positions of the first confined states in QW and QD parts of structures. Arrows indicate most important recombination and carrier transfer channels.

In order to better visualise the design of the investigated TI structures, Fig. 1 presents the sketch of its core part, i.e. the conduction and valence band profiles for four investigated samples, with different QW widths, together with the energies of the first confined hole and electron states in both parts of the structure. It also schematically shows carrier transfer and recombination channels. Although the figure is not in exact scale (the energy distances between consecutive levels are exaggerated to increase its clarity), it is close to real situation, according to the calculations described in the Method section. The ground state of the entire system is positioned in the QD, and the lowest lying levels in the QW are close in energy for the sample with the widest 6 nm well. When the QW gets narrower that distance increases. The influence of this energy separation, controlled by the QW width, is investigated by probing the thermal quenching of PL.

**Figure 2:**
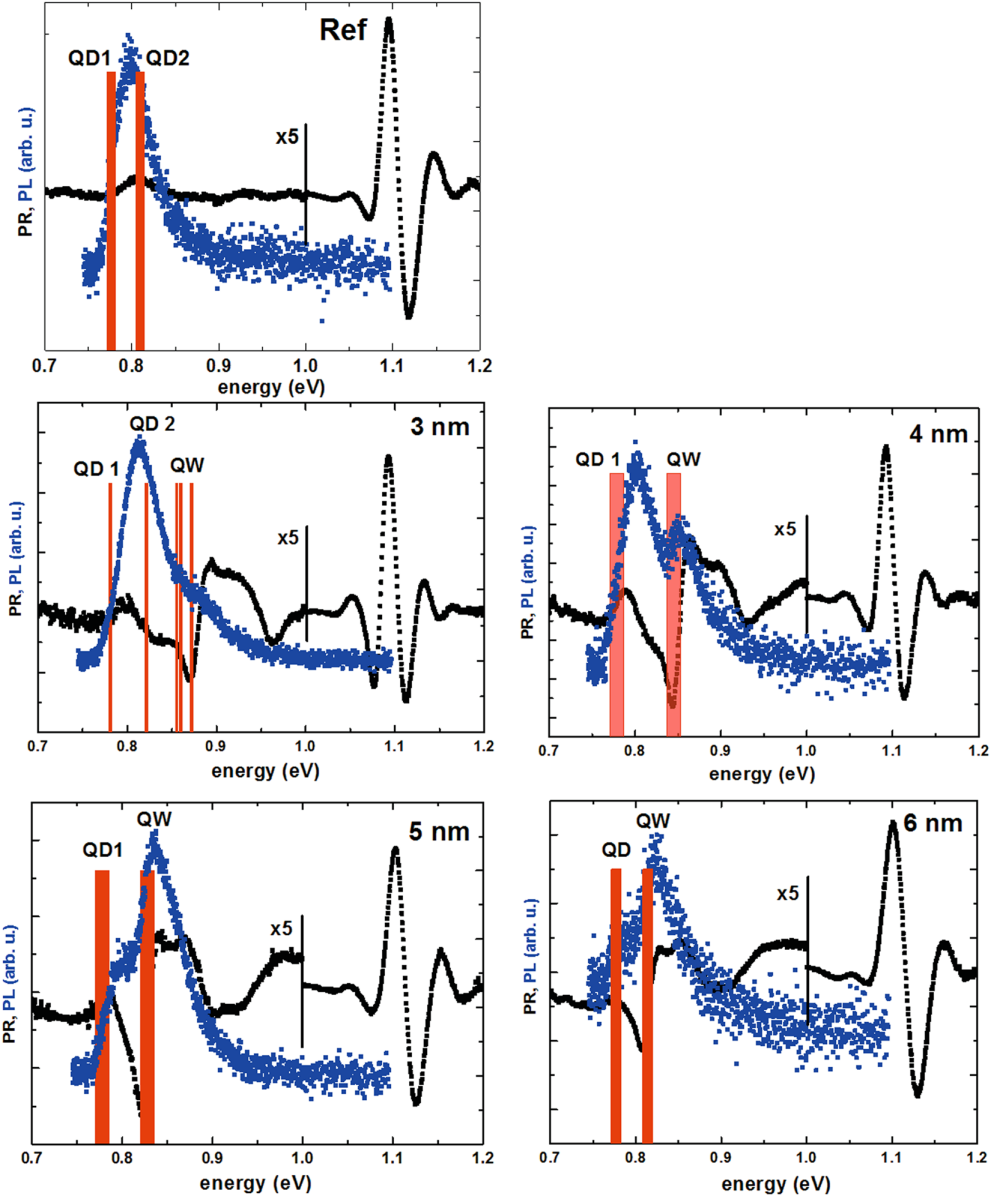
Room temperature PR (black dots) and low excitation power PL (blue dots) spectra for the (**a**) reference and (**b**–**e**) TI injection structures with QW widths of 3 to 6 nm, respectively. Red bars indicate energies of optical transitions.

Complementary optical spectroscopy techniques, i.e. PL, which probes the emission properties; and photoreflectance (PR), a modulation absorption-like technique, are employed to observe optical transitions occurring in the investigated structures at room temperature, with low excitation power densities (see Method section for details). The results are plotted in Fig. 2. PL spectrum for the reference structure (without a QW injector, Fig. 1(a)) shows one Gaussian peak related mainly to the ground state QD emission of the entire inhomogeneous ensemble, centred around 0.8 eV (1.55 μm), as targeted, however, some contribution from the QD excited states cannot be fully excluded at room temperature. The PR spectrum shows a strong feature related to the band gap of InGaAlAs barrier at 1.1 eV energy and a much weaker transition on the low energy side, coinciding with the PL peak. A weak PR response from QDs’ layers is typical due to small total absorption of dots, which translates into small modulated reflectivity response. Vertical bars present calculated energies of optical transitions, showing a good agreement with measurements. Optical spectra of TI structures (Fig. 1(b–e)) are much more complex. The ground state transition in the QD layer, visible both in PL and PR spectra, slightly shifts towards lower energy with the increasing QW width due to the effective broadening of the potential. A stronger PR feature can be also seen, corresponding to the second PL peak, attributed to the QW ground state transition. Its energy, as expected, decreases with increasing QW width, at the same time decreasing the separation between the QW and QD related transitions. It is accompanied by the relative change of their emission intensities, with QW one getting progressively stronger. Again, the calculated transition energies seem to well reproduce the experimental results, especially in the case of the QW transition.

### Photoluminescence quenching

**Figure 3:**
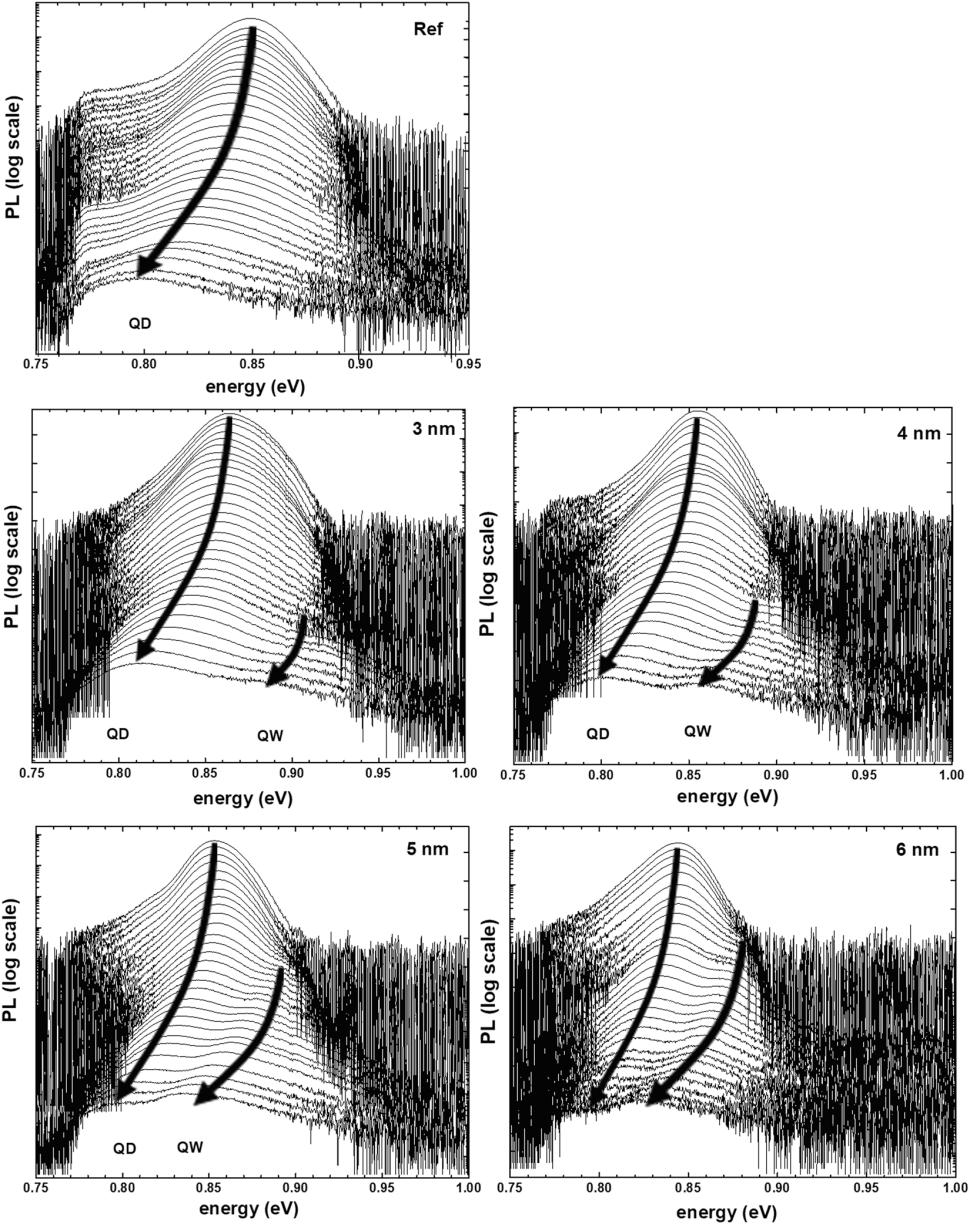
Temperature dependence of PL for (**a**) reference and (**b**–**e**) TI injection structures with QW widths of 3 to 6 nm, respectively. Arrows indicate the position of emission peak maximum at a given temperature.

With the identified hierarchy of optical transitions observed in the investigated samples it is now possible to analyse the effect of temperature on the emission efficiency as a function of the QW and QD levels separation, controlled by the QW width, and driving changes in the carrier transfer between both parts of the system. Photolumienescence vs temperature for all the sample is measured in the temperature range between 13 and 300 K, at the same experimental conditions, to make possible a direct comparison between PL intensities of different samples. The applied excitation power is low (approx. 50 μW) to avoid emission from QD excited states. The obtained results are gathered in Fig. 3, presented in a log scale.

**Figure 4:**
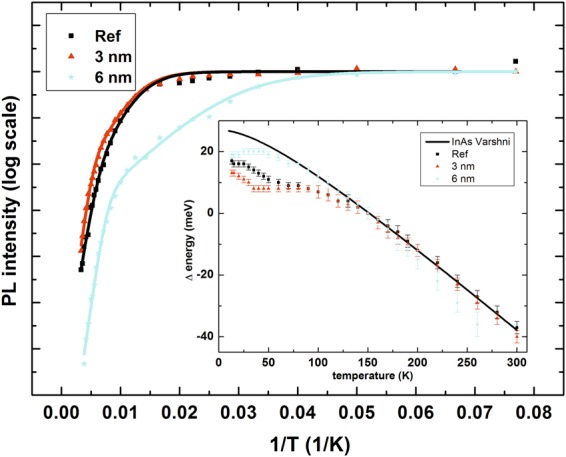
Arrhenius plot of temperature dependence of emission peak areas taken from Gaussian fits to PL spectra for QD transition. (Inset) Temperature dependence of changes in PL peak energies compared with Varshni curve for InAs material, with 150 K taken as a point of reference.

The reference sample shows one peak, shifting to lower energy with increasing temperature. To analyse the temperature dependence of PL peak energies in more details, the changes of the energies relative to the emission energies at 150 K (chosen as a point where any thermal redistribution processes between the dots should be over) are plotted in an inset of Fig. 4. As a reference, a Varshni curve is supplied, with parameters for the band gap energy of InAs material^27^, which constitutes the QDs and thus is the most important factor for the changes of QD related emission energies. Above 150 K, the Varshni curve and the plots for the reference and 3 nm TI structure are in excellent agreement, while the plot for 6 nm TI structure shows slight deviation. It can be explained by a strong influence of the escape of carrier to the QW and its emission, which (i) does not affect the whole dot population in the same way (transfer of carriers from smaller dots is more efficient) and (ii) decreases the accuracy of energy determination for QD emission. Moreover, the ground state energy for the 6 nm TI structure is influenced by the changes in the InGaAs QW bandgap, having larger temperature coefficient than InAs. At lower temperatures, the discrepancies between Varshni curve and determined PL positions are evident, which is typical for QD structures and is related to thermal redistribution of carriers within QD population, affected by the presence of the QW.

In the case of TI structures, beside the QD related transition showing similar the behaviour to the reference structures, an additional peak can be seen, at higher energies. As established above, it can be attributed to the transition between states confined in the QW. This peak appears at different temperatures, depending on the QW width. For the narrowest 3 nm QW it is seen only above 160 K, while for the widest 6 nm QW it can be observed already at 40 K, indicating strong dependence of carrier escape rate from QD to QW on the energy difference between their respective ground states. In order to perform more quantitative analysis of the temperature PL quenching results, the PL spectra are fitted by Gaussian line shapes typical for the emission from an inhomogeneous system. At low temperatures only one peak is used, but when the asymmetry of the peak becomes evident, the second one is added to the fitting model to include the QW emission. The obtained results for QD peak areas for the reference and TI samples with 3 and 6 nm wide QWs are shown in Fig. 4, in Arrhenius type plot, with PL intensity normalised to low temperature value. When comparing the results between the samples, it can be observed that the high temperature parts of Arrhenius plots are similar, but in the intermediate temperature range considerable discrepancies between curves are evident.

The curves from Fig. 4 are fitted by the typical Arrhenius dependence of ln(I) vs (1/T), defined by Equation (1) ^28^:

1 $$lnI=ln\frac{{I}_{0}}{1+{C}_{1}{e}^{-\frac{{E}_{1}}{k}(\frac{1}{T})}+{C}_{2}{e}^{-\frac{{E}_{2}}{k}(\frac{1}{T})}}$$

including two carrier escape processes, with energies E_1_ and E_2_, amplitudes C_1_ and C_2_; and I_0_ indicating PL intensity at 0 K. The obtained results are shown in Table 1. It is worth mentioning here that the activation energies determined by the fitting cannot be directly related to energy differences in the energy structure since they are actually averaged out over all the dots, having different contributions to the total escape processes. The number obtained from the fitting is an effective energy of a given process and it can be interpreted when compared between the samples, indicating overall efficiency of carrier escape from a given population of dots. Such a number will represent the losses, which limit the efficiency of radiative recombination from the QD ground state of the sample.

**Table 1:** Activation energies of escape processes determined from Arrhenius fits to PL temperature quenches. The uncertainties are determined from the fitting procedure.

Sample	E_1_ (meV)	E_2_ (meV)
Reference	30 ± 5	96 ± 10
3 nm QW	30 ± 2	134 ± 9
4 nm QW	18 ± 2	102 ± 5
5 nm QW	14 ± 2	132 ± 7
6 nm QW	13 ± 1	100 ± 5

The model with two carrier escape channel reproduces the experimental spectra considerably well. The higher energies determined for all the samples are grouped around 100 or 130 meV. The Table 1 shows the uncertainty of their determination based on the fitting procedure, however, the total uncertainty can be expected to be even larger, because the activation energy for high energy process is determined from high temperature PL spectra, with low PL intensity and additionally disturbed by the presence of the second, QW related emission peak, which overlaps with QD emission. Moreover, the remark about the influence of QD distribution is valid here. Finally, this process is attributed to the thermal escape of electrons from the lowest energy electron state in the QDs either directly to the electron state in the WL, whose calculated energy is ~100 meV higher, or with the assistance of optical phonons, which for III-V materials have the energy of around 30 meV and thus account for the increased E_2_. In the case of the lower activation energy E_1_, the reference sample and TI sample with 3 nm QW share the same activation energy of 30 meV. As calculations show, this number may be attributed to the energy distance between the first two electron levels confined in the QDs, indicating that for the narrowest QW, the QD ground state is well isolated from the QW influence, with at least one higher energy electron level in QD lying below the first QW electron level and carrier escape into the injector QW states is not efficient, as confirmed also by the fact that the QW emission in this sample occurs only for temperatures above 160 K. For the sample with 4 nm QW, the activation energy equals to 18 meV, already below the energy distance between electron levels confined in the QD. It is thus attributed to the direct escape of electrons from the QD ground state to the QW electron state. For the further increasing QW width, this energy decreases (QW electron state moves closer to the QD electron ground state), indicating higher efficiency of out-tunneling processes of electrons from the QD population to the QW layer. It correlates well with decreasing temperature of QW emission onset seen in Fig. 3.

**Figure 5:**
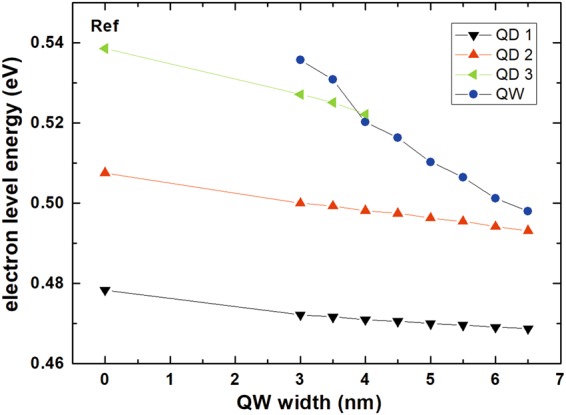
The first three electron levels confined in the dots and the lowest electron level confined in the injector QW as a function of QW width. 0 nm QW width denotes the reference sample without the QW.

**Figure 6:**
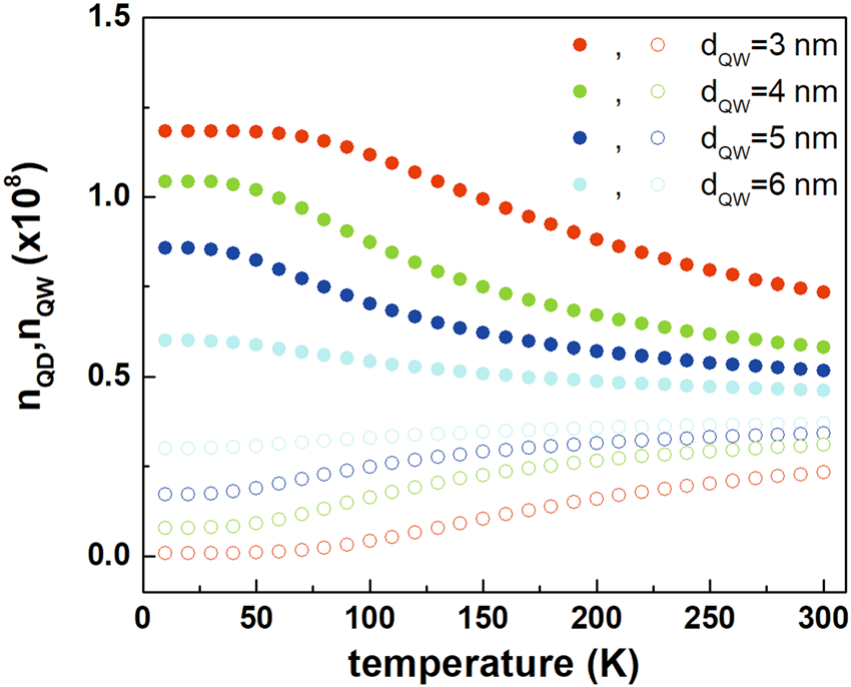
Temperature dependence of QW and QD ground state occupations for all the investigated TI structures, calculated for the continuous pumping of the QW at the level of 10^8^ carriers.

The processes described above can be explained by looking at the confined levels energy structure for the whole system, given in Fig. 5. A remark concerning calculation accuracy is required here. The accuracy of material parameters for quaternary InGaAlAs material and the incomplete knowledge on the exact geometry and chemical content (potential Al and As diffusion into the dots) make it impossible to calculate the absolute energy of levels with the accuracy on the order of meV. However, the tendencies should be reasonably well preserved in the simulations. Therefore, it can be assumed that the level separation for the dots is indeed around 30 meV and their energies should decrease with QW width increase due to the increase in the effective potential width. Also the dependence of the injector level on the QW width is correct. However, the absolute relations between those two groups of level are difficult to capture, mainly because of the finite accuracy of the value of conduction band discontinuity between InGaAlAs, GaAlAs and InAs materials, which shifts their relative positions. The comparison between calculated and measured transition energy shown in Fig. 2 suggests that the electron levels in an average dot should be 15–20 meV higher than shown in Fig. 6. It is also necessary to bear in mind that dots have certain distribution of sizes and compositions, meaning that the relative position of levels in a given dot and QW are also differing from dot to dot. Having taken it all into account it is nevertheless possible to understand the experimental results. For the reference sample and 3 nm TI sample at least two lowest electron levels are confined in the dots, explaining their similar PL quenching characteristics. For wider QWs, the first QW level goes below the second QD level and the effective carrier escape activation energy gets smaller than 30 meV, reflecting the difference between these two levels. Concerning transfer selectivity, efficient transfer occurs when levels in QD and QW are close to resonance, when absorption or emission of acoustic phonons can satisfy the energy conservation, as is the case for 3 nm TI sample between the third QD level and QW level; or when they differ by lateral optical phonon energy, which is ~30 meV for this material system. Therefore when the injector level is ~15 meV above the QD ground state, as seems to be the case for 5 and 6 nm QW, only some of the dots can be efficiently supplied with carriers, because the excess energy is too low for optical phonon and too large for acoustic ones. It explains the decreased broadening of PL peak for these two samples. Finally, it is worth noting that in Fig. 6 there can be observed an anti-crossing between the QW level and the third QD level, however the strength of the coupling it denotes is weak, which is expected due to very different symmetries of respective wave functions.

### Rate equation model

In order to relate observed temperature dependence of PL in a more qualitative way, a simple rate equation model is proposed, with minimum number of parameters. It is focused on the electron relaxation process, assuming that holes undergo rapid and instantaneous relaxation to the bottom of the valence band. The model introduces two coupled levels as presented in Eqs (2 and 3).

2 $$\frac{d{n}_{QW}}{dt}=-\frac{{n}_{QW}}{{\tau }_{rec}^{QW}}-\frac{{n}_{QW}}{{\tau }_{TUN}(T)}(1-\frac{{n}_{QD}}{2{N}_{QD}})+\frac{{n}_{QD}}{{\tau }_{TUN}(T)}{e}^{-\frac{{\rm{\Delta }}E}{{k}_{B}T}}$$

3 $$\frac{d{n}_{QD}}{dt}=-\frac{{n}_{QD}}{{\tau }_{rec}^{QD}}+\frac{{n}_{QW}}{{\tau }_{TUN}(T)}(1-\frac{{n}_{QD}}{2{N}_{QD}})-\frac{{n}_{QD}}{{\tau }_{TUN}(T)}{e}^{-\frac{{\rm{\Delta }}E}{{k}_{B}T}}.$$

Equation (2) describes temporal changes in electron population ${n}_{QW}(t)\,\,$among its large reservoir settled on the QW side, whereas Eq. (2) is responsible for electron kinetics ${n}_{QD}(t)\,\,$among the ensemble of quantum dot ground states. ${N}_{QD}\,\,$is the density of QDs in the layer which defines also the number of available fundamental states in the ensemble of dots to *2N*_*QD*_ (including spin). The ${\tau }_{rec}^{QW}$, ${\tau }_{rec}^{QD}$, and ${\tau }_{TUN}(T)$ are temperature insensitive QW and QD radiative lifetimes, and temperature dependent average transfer time (Eq. (4)) between the electron reservoir and QDs.

4 $$\frac{1}{{\tau }_{TUN}(T)}=\frac{({N}_{Ph}(T)+1)}{{\tau }_{TUN}((T=0\,K)}$$

It is assumed here that the carrier transfer is controlled rather by phonon-mediated than Auger-type processes and thus the ${\tau }_{TUN}(T)$ is contributed by the phonon occupation number ${N}_{Ph}(T)={(\exp (({E}_{ph})/{k}_{B}{\rm{T}})-1)}^{-1}$, where ${\tau }_{TUN}(T=0\,K)$ is the carrier transfer time at low temperatures, *E*_*ph*_ is the average phonon energy, and *k*_*B*_ is the Boltzmann constant. Last terms in Eqs (2 and 3) account for the temperature activated back transfer process from the QDs to the QW reservoir that depends on the energy difference ΔE between both states. The coupled rate equation system has been solved for the steady-state condition at different temperatures and for experimentally obtained parameters summarized in Table 2.

**Table 2:** Parameters used for the rate equation model.

QW thickness d_QW_(nm)	${{\boldsymbol{\tau }}}_{{\bf{r}}{\bf{e}}{\bf{c}}}^{{\bf{Q}}{\bf{D}}}$ (ns)	${{\boldsymbol{\tau }}}_{{\bf{r}}{\bf{e}}{\bf{c}}}^{{\bf{Q}}{\bf{W}}}$ (ns)	${\boldsymbol{\Delta }}{\bf{E}}{\boldsymbol{=}}{{\bf{E}}}_{{\bf{p}}{\bf{h}}}$ (meV)	${{\boldsymbol{\tau }}}_{{\bf{T}}{\bf{U}}{\bf{N}}}{\boldsymbol{(}}{\bf{T}}{\boldsymbol{=}}{\bf{0}}\,{\bf{K}}{\boldsymbol{)}}$ (ps)
3	1.2	0.6	30	78
4			18	90
5			14	240
6			13	560

The surface density of quantum dots *N*_*QD*_ has been deduced from the growth calibration and structural characterization to be of the order of 1 × 10^11^ cm^−2^. The ${\tau }_{TUN}(T=0\,K)$ and ${\tau }_{rec}^{QD}$ have been obtained from the time-resolved photoluminescence experiment at low temperature through analysis of the PL rise and decay profiles, respectively, ΔE and *E*_*ph*_ are directly taken from Table 1 for the first activation process that corresponds to the most efficient carrier relaxation/activation channels. The only parameter which had to be assumed is ${\tau }_{rec}^{QW}$. In our model ${\tau }_{rec}^{QW}$ = 0.6 ns, which is in a reasonable agreement with values found in the literature^8,29,30^ for InGaAs/InP QWs. The obtained temperature dependence of level occupations as a function of temperature is plotted in Fig. 6 for all the investigated TI structures. Although the emission at low temperatures is non-zero for the samples with wider QW, contrary to the experiment, still the existence of a threshold temperature, where QW emission starts to increase, is evident and in a reasonable agreement with experimental observations. This discrepancy may be attributed to the limitations of the simplified model employed here (for example the omission of QW related non-radiative recombination channels).

### Discussion – influence of tunnel injection on emission

**Figure 7:**
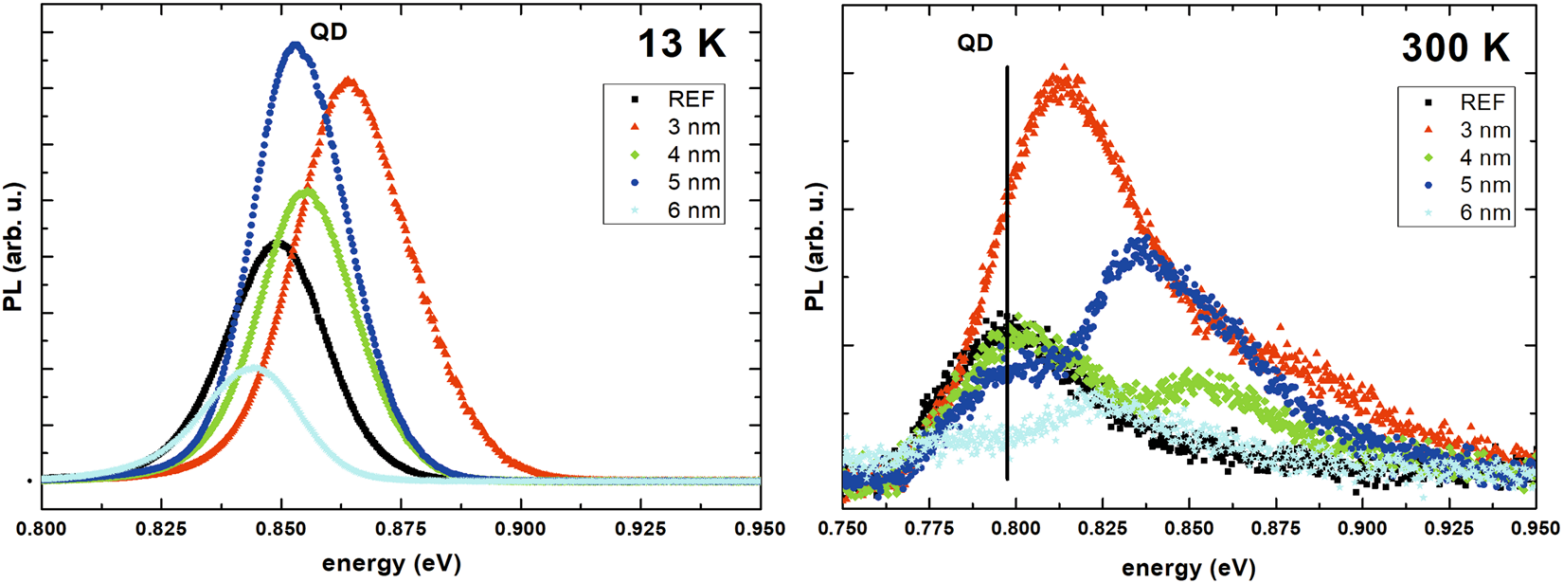
PL spectra for all the samples measured at (**a**) 13 K and (**b**) room temperature.

The intensity of emission from the QDs is governed by: (i) the oscillator strength of the ground state transition, which should be very similar for all the samples due to identical dots growth conditions and the resulting wave functions’ overlap between the lowest energy electron and hole levels; (ii) the absorption of the excitation light, which again is comparable between the structures, being dominated by the identical wide InGaAlAs barriers present in all of them; (iii) carriers’ kinetics, i.e. the efficiency of the competing processes of carrier capture and escape, which are expected to depend on the coupling between the QW and QDs and details of the electronic structure. Figure 7(a) shows the PL spectra at 15 K. It can be seen that the intensities of emission from TI structures with 3, 4 and 5 nm QW are higher than for the reference structure, showing positive impact of the QW even when phonon population is very low. For the sample with 6 nm QW the emission intensity is lower than for the reference sample, which may be due to the fact that some of the larger dots in this sample become actually of type II, because the lowest energy hole level in the QW is above the QD hole level. At elevated temperatures the situation changes, due to the activation of transfer channels both to and from the dots. Finally, at room temperature (Fig. 7(b)), the intensity of PL from the sample with the narrowest QW becomes the highest, because the escape process is the least efficient, as shown above by the rate equation model. To conclude, due to the narrow 2 nm barrier in the investigated samples the transfer of carriers from the QW to the dots is very effective, even if for the narrow QW it occurs via the excited states in the dots. For elevated temperatures, the back-transfer from the dots to the QW starts to play a role, resulting in the radiative recombination into the QW and activating possible additional channels of non-radiative carrier losses, which limits the emission intensity of the QD ground state of the whole system. However, for the narrow enough QW this process does not dominate and the emission intensity of the TI structures may be stronger than for the reference QDs at room temperature, making the TI system suitable for active part of efficient telecom lasers.

### Summary

Tunnel injection structures based on the InAs/InP quantum dots, with the injector InGaAlAs QW of varying thicknesses, have been investigated by means of optical spectroscopy, with special focus on temperature dependence of PL intensity. Room temperature photoreflectance and photoluminescence spectra, supported by the calculations in an 8-band k∙p model, have been used in order to identify the optical transitions by attributing them to the transitions between particular confined levels in the hybrid 0D-2D system. Analysis of Arrhenius plots has allowed determining carrier escape channels from the dots, pointing at the importance of carrier escape into the QW. It has been demonstrated that for narrower QWs the escape process is less efficient than carrier supply via the QW (due to the thin barrier) when compared to wider injector wells, resulting in improved emission intensity at room temperature. This finding is supported by the rate equation model, assuming temperature dependent carrier transfer between QW and QDs. It demonstrates that with a careful design of energy level structure, a hybrid QW/QD system grown on InP substrates used as an active region of a QD-based laser, may offer improved performance for telecommunication applications.

## Methods

### Sample design

All the samples are grown by Molecular Beam Epitaxy on (100) oriented nominally undoped InP substrates. On top of an InP buffer layer, a 100 nm quaternary In_0.53_Ga_0.23_Al_0.24_As layer is deposited, followed by an In_0.53_Ga_0.47_As QW with thicknesses of 3, 4, 5 and 6 nm, varying between different samples. The QW is then separated by a 2 nm thin In_0.53_Ga_0.23_Al_0.24_As tunnelling barrier from InAs QDs. The deposition of 4.3 monolayers (ML) of InAs material on In_0.53_Ga_0.23_Al_0.24_As barrier results in Stranski-Krastanow growth of self-assembled QDs on a thin WL (3 ML in this case^31^). The use of the cracked version of arsenic (As_2_), instead of commonly used As_4_, affects the diffusion on the surface, leading to the nucleation of almost in-plane symmetric QDs, in spite of natural tendency to strong nanostructure elongation in the MBE growth for this material system^32^. For reference, a QD sample without QW is also grown. The thicknesses of respective layers are controlled by the growth parameters which were calibrated for thick InGaAs or InGaAlAs layers. The average dot sizes are determined from cross-sectional transmission electron microscope to be 2.5 × 16.5 × 33 nm, which gives a total volume considerably smaller than in quantum dashes and thus providing larger separation between confined levels, which is beneficial for the design of TI structures. The dots are covered by another 100 nm thick In_0.53_Ga_0.23_Al_0.24_As layer. The whole structure is capped with a 10 nm InP layer to prevent Al oxidation. All materials, except for InAs, are lattice matched to the InP substrate.

### Optical measurements

Room temperature PR experiment is used to determine electronic structure of investigated samples by identifying relevant optical transition energies. It uses a halogen lamp as a broadband light source and a 630 nm line from a semiconductor diode laser for modulation. The light reflected off a sample is dispersed by a monochromator with 0.3 m focal length and detected by an InGaAs p-i-n photodiode. The PR measures the changes in the reflectivity spectrum upon photo-modulation, resulting in a derivative-like response, with clear signatures of optical transitions^33^. For PL experiment the samples are mounted on a cold finger in a close cycle helium refrigerator, in the temperature range from 13 to 300 K (0.5 K temperature setting accuracy), provided by a heater. The non-resonant excitation is provided by a mode-locked Ti:Sapphire laser, at a wavelength of 830 nm (energy of 1.5 eV), above bandgaps of all layers in the investigated samples, with weak average excitation power of 50 µW (~7 W/cm^2^), chosen in such a way as to just excite emission up to room temperature. The obtained PL signal is dispersed by a 0.5 m focal length monochromator and recorded with an InGaAs linear array detector.

### Calculations

The calculations are used to reproduce the energy level structure of states confined in the system and interpret the results of optical measurements. In order to include the potential of quantum dots, the 3D eight-band k⋅p model is used, including strain fields^34^, piezoelectric effects, and spin-orbit interaction^35,36^. All equations are solved numerically, using the finite-difference method^35–37^. Material parameters are taken after refs^38,39^. The QDs are modelled as lens-shaped. In order to obtain a better agreement between the simulations and experiment, 3% of Al and Ga atoms are added to nominally pure InAs QD material, which can be explained by the intermixing during the growth^40^. In order to take into account the existence of a 1D and 3D confinement potential in the same calculations, periodic boundary conditions are imposed, enabling proper computation of the QW states in the 3D model.
